# Estimation of Nonlinear Adsorption Isotherms in Gradient Elution RP-LC of Peptides in the Presence of an Adsorbing Additive

**DOI:** 10.1007/s10337-017-3298-y

**Published:** 2017-03-28

**Authors:** Dennis Åsberg, Marek Leśko, Tomas Leek, Jörgen Samuelsson, Krzysztof Kaczmarski, Torgny Fornstedt

**Affiliations:** 1 Department of Engineering and Chemical Sciences, Karlstad University, 651 88 Karlstad, Sweden; 2 Department of Chemical and Process Engineering, Rzeszów University of Technology, 359 59 Rzeszów, Poland; 3 Respiratory, Inflammation and Autoimmunity, Innovative Medicines and Early Development Biotech Unit, AstraZeneca, 431 83 Mölndal, Sweden

**Keywords:** Liquid chromatography, Peptide separation, Charged surface hybrid, Adsorption isotherm, Gradient elution

## Abstract

**Abstract:**

In electrostatic repulsive interaction chromatography, using a charged surface hybrid sorbent carrying positive charges can improve the peak shape of peptides in reversed-phase liquid chromatography (RP-LC), especially in overloaded conditions, compared with standard C_18_ sorbents. However, the positive surface charges can interact with anionic additives commonly used in peptide separations, e.g., trifluoroacetic acid (TFA), complicating adsorption isotherm estimation. We investigated how the competition for available adsorption sites between TFA and two peptides influenced the adsorption isotherm in gradient elution. A model accounting for the competition with TFA was compared with a model neglecting TFA adsorption. We found that the two models predicted elution profiles with the same accuracy. We also found that the adsorption isotherms were extremely similar in shape, leading to the conclusion that neglecting the competition with TFA is a valid approximation enabling faster and more robust adsorption isotherm estimation for the studied type of sorbent.

**Graphical abstract:**

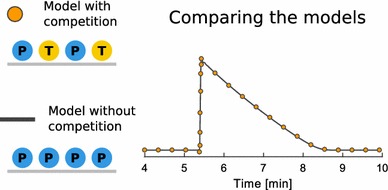

## Introduction

Therapeutic peptides are an important group of biopharmaceuticals and purification with preparative reversed-phase liquid chromatography (RP-LC) is often necessary during manufacturing in order to obtain sufficient purity [1]. Currently, there is a trend in the biopharmaceutical industry towards improving process understanding and replacing statistical and empirical correlations with mechanistic models [2]. Adsorption isotherm determination is an essential step in the mechanistic modeling of preparative LC and is preferably performed using the inverse method (IM) since it requires little experimental work and small amounts of substance [1, 3, 4]. Recently, we extended the IM to estimation of adsorption isotherm parameters directly from overloaded profiles obtained in gradient elution [3–6]. The IM had previously only been used in isocratic elution and it was then cumbersome to determine isotherms for gradient elution since experiments had to be performed at multiple modifier levels in isocratic elution [7].

In general, the retention of most peptides is sensitive to the fraction of organic modifier in the eluent and to the eluent pH, so they are often separated via gradient elution at low pH [8]. An ion-pair reagent, such as trifluoroacetic acid (TFA), is commonly added to adjust the pH and increase the peptide retention, since peptides often have at least one positive charge at low pH. Positively charged peptides can exhibit severe tailing, especially in overloaded conditions, on standard C_18_ stationary phases [9]. To address this, a charged surface hybrid (CSH) C_18_ stationary phase that contains a small number of positively charged groups can be used. However, anionic ion-pairing reagents such as TFA adsorb on CSH stationary phases, thereby potentially competing with the peptide for adsorption. This complicates the determination of peptide adsorption isotherms, since the adsorption isotherm for the ion-pairing reagent must then be taken into account.

This study extends our investigation of nonlinear adsorption isotherm determination with the IM in gradient elution [3–6] to the presence of an adsorbing additive. To this end, the adsorption isotherms on CSH stationary phases of two endogenous opioid peptides in the presence of TFA were determined in gradient elution. Two models were established, one taking the competition for adsorption sites from TFA into account and the other neglecting it. The adsorption isotherms from the two models were compared and their ability to predict elution profiles was evaluated. This study also demonstrates the use of the IM in a practical case, i.e., peptide purification, where gradient elution is routinely employed.

## Theory

The equilibrium-dispersive (ED) model describes the mass balance in the chromatographic column for component *i* [1]:

1 $$ \frac{{\partial C_{i} }}{\partial t} + F\frac{{\partial q_{i} }}{\partial t} + w\frac{{\partial C_{i} }}{\partial x} = D_{\text{a}} \frac{{\partial^{2} C_{i} }}{{\partial x^{2} }}, $$

where *F* = (1 − *ε*_t_)/*ε*_t_ is the phase ratio, *ε*_t_ is the total porosity, *w* is the interstitial mobile phase velocity, *D*_a_ is the apparent dispersion coefficient, *t* and *x* are the time and length coordinates in the column, and *C* and *q* are the local mobile and stationary phase solute concentrations, respectively. Danckwerts-type boundary conditions [1] were used at the column inlet and outlet coupled with experimentally obtained injection profiles for each injection volume. The gradient is then described by the inlet condition for the organic modifier:

2 $$ \varphi \left( t \right) = \left\{ \begin{gathered}   \varphi _{0} {\mkern 1mu} \quad \quad \quad \quad \quad \,\,\,0 \le t < t_{{\text{p}}}  \hfill \\   \varphi _{0}  + \beta \left( {t - t_{p} } \right){\mkern 1mu} \quad t_{{\text{p}}}  \le t < t_{{\text{p}}}  + t_{{\text{g}}}  \hfill \\   \varphi _{0}  + \Delta \varphi \quad \quad \quad \,\,t_{{\text{p}}}  + t_{{\text{g}}}  \le t \hfill \\  \end{gathered}  \right., $$

where *φ*_0_ is the fraction of organic modifier in the mobile phase at the beginning of the gradient, *t*_p_ is the time when the gradient reaches the column inlet, *β* = Δ*φ*/*t*_g_ is the slope of the gradient with Δ*φ* being the change in modifier fraction between the beginning and end of the gradient, and *t*_g_ is the duration of the gradient. In RP-LC, linear solvent strength (LSS) theory [10] can be used to modify the competitive Langmuir adsorption isotherm to incorporate the organic modifier dependence, which then becomes

3 $$ {q_{\text{P}} \left( {C_{\text{P}} ,C_{\text{T}} ,\varphi } \right) = \frac{{a_{\text{P}} {\text{e}}^{{ - S_{\text{a,P}} \varphi }} C_{P} }}{{1 + K_{\text{P}} {\text{e}}^{{ - S_{\text{K,P}} \varphi }} C_{\text{P}} + K_{\text{T}} {\text{e}}^{{ - S_{\text{K,T}} \varphi }} C_{\text{T}} }}}, $$

where *a* is the Henry constant and *K* the association equilibrium constant in pure water, while *S* is a parameter describing the organic modifier dependence. Subscript P denotes the peptide and subscript T denotes TFA.

## Materials and Methods

### Chemicals

The peptides leu-enkephalin (LeuEnk; CAS #58822-25-6) and met-enkephalin (MetEnk; CAS #58569-55-4) were used as solutes. LeuEnk (93%) was purchased from Bachem (Bubendorf, Switzerland) and MetEnk (77%) from Alfa Aesar (Karlsruhe, Germany). Trifluoroacetic acid (≥99.0%) was used as an ion-pairing reagent and purchased from SigmaAldrich (St. Louis, MO, USA). The mobile phase consisted of gradient-grade acetonitrile from VWR (Radnor, PA, USA) and water with a conductivity of 18.2 MΩ cm from a Milli-Q Plus 185 water purification system from Merck Millipore (Darmstadt, Germany).

### Instrumentation

An Acquity H-Class Bio chromatograph from Waters (Milford, MA, USA) with a quaternary solvent manager, an autosampler with a 50-µL extension loop, a column oven, and a PDA detector was used. The column temperature was set to 25.0 °C and the flow rate was 0.25 mL min^−1^. The column was an XSelect CSH C_18_, 100 × 2.1 mm column from Waters. The CSH particles were prepared by derivatizing bare bridged ethylene hybrid particles with a weakly basic ionizable silane before the ligand bonding and the end-capping steps [11]. The C_18_ surface coverage was 2.38 µmol m^−2^ and the amine surface coverage was estimated to be approximately 0.02 µmol m^−2^ [11]. For this specific column, the average particle diameter was 4.81 µm and the total porosity was measured by pycnometry to be 0.668 using acetonitrile and dichloromethane.

### Procedure

The adsorption isotherm of TFA was obtained using the perturbation pulse method [1] on four isocratic plateaus: 10.00, 13.75, 17.50, and 25.00% acetonitrile. Perturbations were obtained for each acetonitrile plateau at 12 TFA concentrations in the range of 0–45 mM. The fraction of acetonitrile in the eluent is given as the weight fraction (w/w), so the gradient slope is given as the weight percentage of acetonitrile per minute. To determine the adsorption isotherms of the peptides in gradient elution, overloaded injections were performed in gradient elution at three gradient slopes: 0.25, 1.00, and 2.75% min^−1^. The gradient was linear and ran from 13 to 25% acetonitrile with no isocratic hold. The TFA concentration was held constant at 37.3 mM in both the eluent and diluent. Overloaded injections were performed by injecting 20 and 50 µL of peptide samples containing 17.9 mM LeuEnk or 13.8 mM MetEnk. The two elution profiles used in the IM were 20 µL, 0.25% min^−1^ and 50 µL, 2.75% min^−1^. Calibration was done by direct integration of the 290-nm response [3]. The column efficiency was determined to be 2700. The adsorption isotherm parameters were determined using the IM following the stepwise approach described by Åsberg et al. [3].

## Results and Discussion

**Fig. 1:**
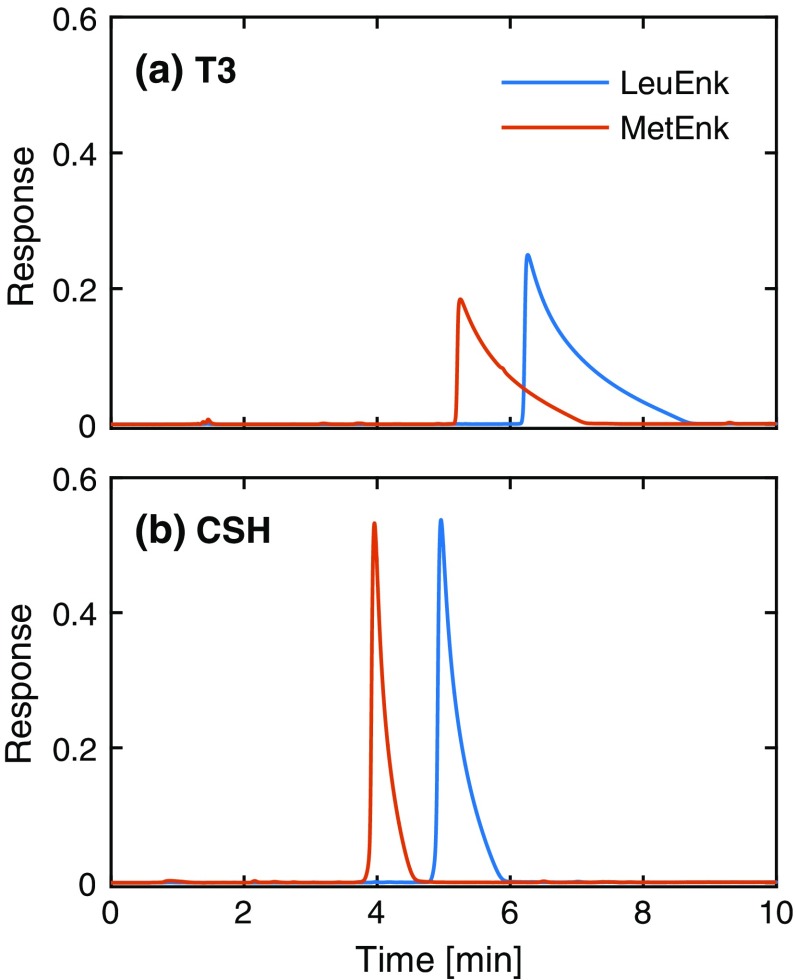
Overloaded elution profiles recorded for the same samples using two different columns: **a** Atlantis T3 C_18_ and **b** XSelect CSH C_18_. The mobile phase was acetonitrile/water with 2.6 mM TFA in gradient elution (10–25% acetonitrile in 10 min); 40 µL of MetEnk (4.4 mM) and LeuEnk (6.7 mM) were injected individually and the chromatograms overlaid. Note the considerably reduced tailing on the CSH column as compared to the T3 column

To demonstrate the gain in peak shape with the CSH column, overloaded elution profiles were recorded on an Atlantis T3 C_18_ column from Waters with the same dimensions and particle size. Figure 1 compares the elution profiles obtained with the two columns. The peaks obtained on the CSH column are much narrower although the same load is used on both columns and baseline separation could be obtained (Fig. 1b). The difference in chromatographic performance is most pronounced at low TFA concentrations as shown in Fig. 1 for 2.6 mM (0.02%, v/v) TFA. In the following experiments, the TFA concentration was increased to 37.3 mM (0.29%, v/v) to include conditions where the peptide concentration is significantly lower than the TFA concentration, i.e., if competition between TFA and the peptide can be neglected at 37.3 mM TFA it can be neglected also at lower TFA concentrations.

### TFA Adsorption Isotherm

The adsorption isotherm slope data obtained from the perturbation pulse experiments at the four acetonitrile fractions were fitted simultaneously to the bi-Langmuir isotherm model extended to gradient elution by applying LSS theory [3]:

4 $$ q(C_{\text{T}} ,\varphi ) = \frac{{a_{{1,{\text{T}}}} {\text{e}}^{{ - S_{{a1,{\text{T}} }}\varphi }} C_{\text{T}} }}{{1 + K_{{1,{\text{T}}}} {\text{e}}^{{ - S_{{K1,{\text{T}}}} \varphi }} C_{\text{T}} }} + \frac{{a_{{2,{\text{T}}}} {\text{e}}^{{ - S_{{a2,{\text{T}} }}\varphi }} C_{\text{T}} }}{{1 + K_{{2,{\text{T}}}} {\text{e}}^{{ - S_{{K2,{\text{T}} }}\varphi }} C_{\text{T}} }}. $$

The rationale for the bi-Langmuir model was that the sorbent surface contains two types of possible interactions with TFA: the hydrophobic interactions with the C_18_ layer (partitioning) and the electrostatic interactions with the positively charged amine groups [11]. The fit was very good with *R* ^2^ = 0.9930 and the numerical parameters were estimated to be

*a*_1,T_ = 1.13, *S*_*a*1,T_ = 3.29, *K*_1,T_ = 13.4 M^−1^, and *S*_*K*1,T_ = 187,

*a*_2,T_ = 6.30, *S*_*a*2,T_ = 3.99, *K*_2,T_ = 408 M^−1^, and *S*_*K*2,T_ = 4.42.

**Fig. 2:**
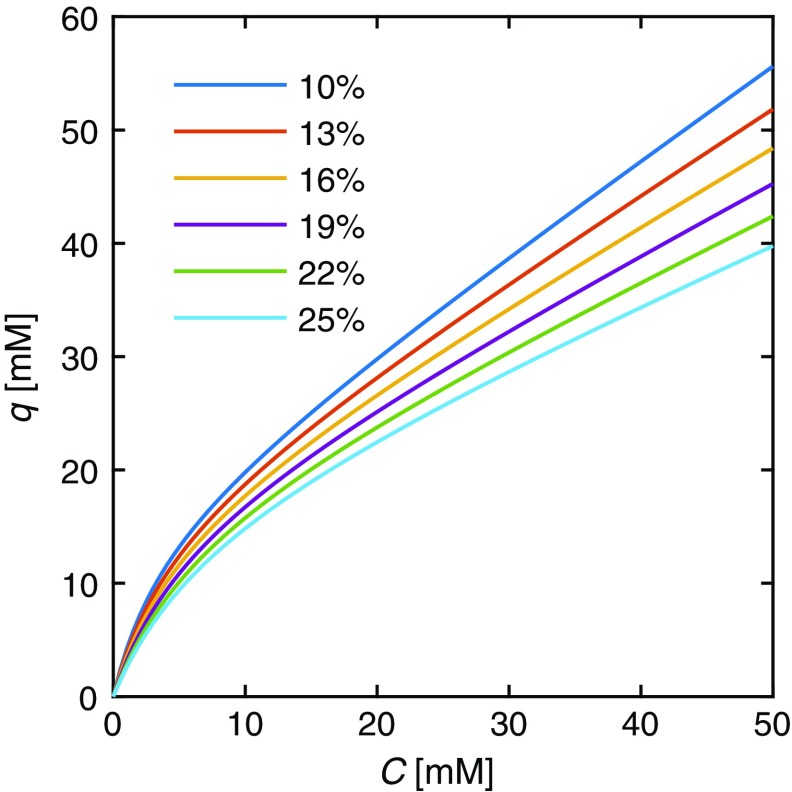
Bi-Langmuir adsorption isotherm, Eq. (4), for TFA on the CSH stationary phase with different fractions of acetonitrile in the mobile phase (given in the legend)

Subscript 1 denotes the site corresponding to adsorption on the C_18_ layer and subscript 2 denotes interactions with the charged groups. Since *K*_1,T_ ≪ *K*_2,T_, the interactions at site 1 are much weaker compared to site 2 and the saturation capacity is higher for site 1. This is expected since the electrostatic interactions are stronger than the hydrophobic ones for a small ionic solute such as TFA and the surface coverage is lower for the charged ligand. The adsorption isotherm for TFA shows an increasing adsorption of TFA on CSH surface with increasing acetonitrile fractions (Fig. 2) and therefore acetonitrile dependence must be taken into account when studying the adsorption isotherm of TFA in gradient elution.

### Peptide Modeling

LeuEnk and MetEnk have a charge of +1 at the studied pH and can therefore be repelled from the positive groups on the CSH stationary phase. The only interaction is therefore with the C_18_ layer and consequently the competitive Langmuir isotherm, Eq. (3), is suitable. Two adsorption isotherm models were evaluated using the inverse method: model A accounts for the competition with TFA for the C_18_ sites and model B neglects the TFA adsorption. Model A uses the values *K*_1,T_ = 13.4 M^−1^ and *S*_*K*1,T_ = 187 for TFA in Eq. (3) obtained from the perturbation pulse experiments, while model B assumes that *K*_1,T_ = 0 and hence no competition with TFA. The resulting models show a large overlap between experimental and calculated chromatograms; 94.1% area overlap for LeuEnk and 95.1% overlap for MetEnk, which is considered very good.

**Fig. 3:**
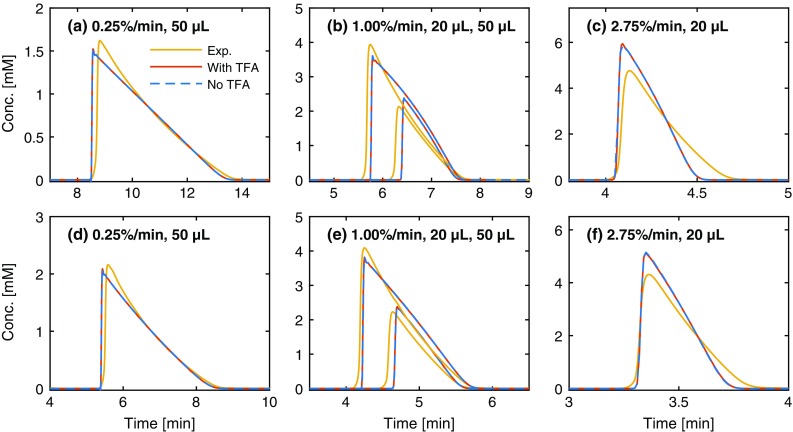
Experimental (Exp.) elution profiles and elution profiles predicted using the two models for LeuEnk (**a**–**c**) and MetEnk (**d**–**f**) at different gradient slopes and injection volumes. “With TFA” denotes model A, which accounts for the competitive adsorption of TFA, and “No TFA” denotes model B, which neglects TFA adsorption. Note the very good agreement between the results of the two models, indicating that neglecting TFA does not alter the predictive power

Regarding process optimization, the model’s ability to predict elution profiles is the most important characteristic, while for process understanding, the adsorption isotherm model per se is most important [5, 12]. To evaluate the models predictive power, four elution profiles were predicted and compared with experimental results, varying both the injection volume and gradient slope for both peptides (Fig. 3). Both models predict identical elution profiles for all conditions, so we conclude that the predictive power of the model does not decrease when the adsorption of TFA is neglected under the studied conditions. The overall predictions are in good agreement with experimental results, considering how sensitive the peptides are to the acetonitrile fraction.

**Fig. 4:**
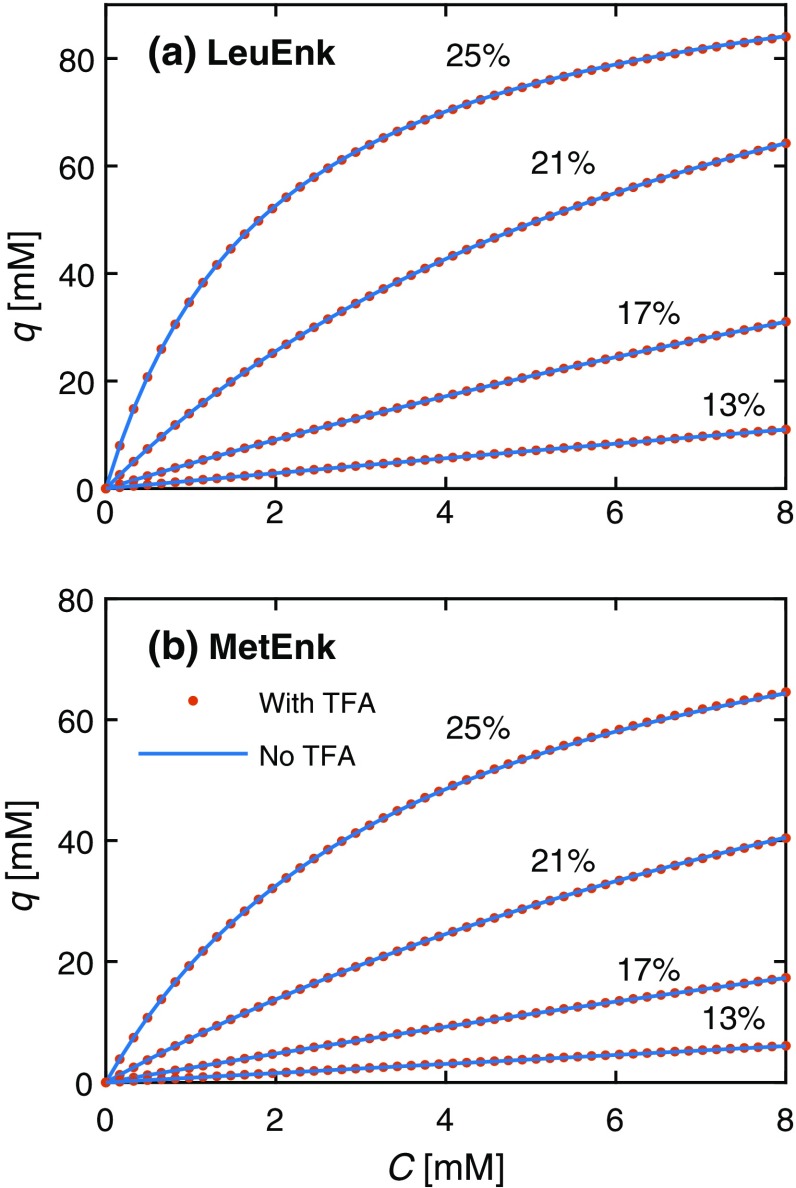
Adsorption isotherms obtained using the two models for **a** LeuEnk and **b** MetEnk at different acetonitrile fractions in the mobile phase. The acetonitrile fractions are given above each isotherm line. “With TFA” denotes model A, which accounts for the competitive adsorption of TFA, and “No TFA” denotes model B, which neglects TFA adsorption. Note the excellent agreement between the two models, indicating that neglecting TFA does not change the shape of the adsorption isotherm

We have previously demonstrated that the estimated isotherm parameters obtained using the inverse method in gradient elution lack physical meaning and should be treated as numerical fitting parameters, although the overall shape of the adsorption isotherm is physically correct [5]. Therefore, the shapes of the adsorption isotherms obtained using the two models are compared in Fig. 4. Excellent agreement was found between model A and B results at all acetonitrile fractions, indicating that neglecting TFA adsorption provides a valid approximation when acquiring adsorption isotherms for such systems. The physical explanation is that TFA adsorbs mainly on the positively charged groups, denoted as site 2, and very weakly on the C_18_-type sites (site 1), while the peptides adsorb strongly on these sites. The competition from TFA therefore has a minor influence on the peptide adsorption mechanism. Being able to neglect the TFA in the estimation of the peptide’s adsorption isotherm has two clear advantages: (1) it decreases the number of required experiments since the TFA adsorption isotherm does not need to be determined and (2) the parameter estimation in the IM becomes more robust, i.e., probability to get stuck in a local minimum decreases, when the number of components decreases.

## Conclusions

We investigated the performance of the inverse method for adsorption isotherm determination in gradient elution in the case of an adsorbing additive by modeling the adsorption of two peptides on a CSH C_18_ stationary phase with TFA in the eluent. We found that TFA competition could be neglected without loss of predictive power or loss of physical relevance of the adsorption isotherm since TFA probably adsorbs mainly on the positively charged ligands rather than on the C_18_ ligands and therefore does not compete with the peptide for the available C_18_ adsorption sites. Being able to neglect the adsorption of TFA reduces the number of experiments and allows the use of a simplified mechanistic model which in turn increases the calculation speed and robustness, which is especially important in process optimization involving competitive adsorption isotherms of three or more peptides [13].
